# Luxation of the Patella on Its Axis

**Published:** 1843-05

**Authors:** Joseph P. Gazzam

**Affiliations:** Pittsburg, Pa.


*Luxation of the Patella on its axis.* By Joseph P. Gazzam, M. D., of Pittsburg, Pa.—Sept. 10*th*, 1842. This evening at 7 o'clock, James, aged 21 years, son of Judge Porter, of Pittsburg, was thrown while wrestling, and immediately found himself unable to rise.

On seeing him about an hour after the accident, I found the patella of the right leg dislocated on its axis, i. e. it was lying on its edge—presenting the posterior face outward, and the anterior face inward—the inner edge resting in the groove between the condyles of the femur.

Flexing the thigh on the pelvis and straightening the leg, I endeavored to replace the bone by pressing its edges in opposite directions, but failing (after repeated trials) I requested that the patient should be brought to town, (the accident happened three miles out of the city), and additioncl advice procured.

At about 12 o'clock the patient was brought to his father's house, where I met Dr. Addison. After repeated unsuccessful attempts at reduction, it was thought well to lessen the tension of the joint by dividing the ligament of the patella. This I did by introducing beneath the skin a narrow-bladed knife, and cutting the ligament close to the tubercle of the tibia. Again we attempted reduction but failed. The patella could be moved on its edge more freely than before the cutting, but resisted all our efforts to replace it.

Dr. Speer was now joined to the consultation. and in accordance with his suggestion the patient was placed erect, a vein opened, and the blood allowed to flow until the approach of syncope. when the efforts at reduction were renewed—but although the patella could be moved on its edge, it could not be lifted out of the groove in which it rested. It was now agreed to let the patient rest for a few hours.

11*th.* At 8 A. M. the consultation was resumed, and it was now proposed to adopt with some modification the plan of Dr. John Watson, of New York, as detailed in the *N. Y. Journ. of Med. Surg.* No. 2, and republished in the *Am. Jour. of Med. Sciences*, vol. 25, p. 252.

The thigh was strongly flexed on the pelvis and the heel elevated. Then the leg was flexed steadily and forcibly on the thigh and suddenly straightened. At the moment of straightening the leg I pressed very strongly against the lower edge of the patella from without, with the head of a door key well wrapped, while Dr. Addison pressed with both thumbs against the upper edge of the bone towards the external condyle. On the fourth trial this manœuvre succeeded, the bone springing into its place with a snap. A cushioned splint was placed behind the knee and secured by a bandage—an evaporating lotion was used, and the patient kept at rest. Recovery was uninterrupted, and the young man has now perfect command of the limb.

To the inexperienced it may seem that I have attached undue importance to this case by reporting it for the medical public; but I have no fear that those who have encountered such a case will think it altogether valueless.—*Amer. Journ. Med. Sci.*

